# Transmissibility of Atypical Scrapie in Ovine Transgenic Mice: Major Effects of Host Prion Protein Expression and Donor Prion Genotype

**DOI:** 10.1371/journal.pone.0007300

**Published:** 2009-10-06

**Authors:** Jean-Noël Arsac, Dominique Bétemps, Eric Morignat, Cécile Féraudet, Anna Bencsik, Denise Aubert, Jacques Grassi, Thierry Baron

**Affiliations:** 1 Agence Française de Sécurité Sanitaire des Aliments, Lyon, France; 2 Commissariat à l'Energie Atomique, Service de Pharmacologie et d'Immunoanalyse, Gif sur Yvette, France; 3 Ecole Normale Supérieure de Lyon, CNRS/INRA, Lyon, France; Universidade Federal do Rio de Janeiro (UFRJ), Instituto de Biofísica da UFRJ, Brazil

## Abstract

Atypical scrapie or Nor98 has been identified as a transmissible spongiform encephalopathy (TSE) that is clearly distinguishable from classical scrapie and BSE, notably regarding the biochemical features of the protease-resistant prion protein PrP^res^ and the genetic factors involved in susceptibility to the disease. In this study we transmitted the disease from a series of 12 French atypical scrapie isolates in a transgenic mouse model (TgOvPrP4) overexpressing in the brain ∼0.25, 1.5 or 6× the levels of the PrP^ARQ^ ovine prion protein under the control of the neuron-specific enolase promoter. We used an approach based on serum PrP^c^ measurements that appeared to reflect the different PrP^c^ expression levels in the central nervous system. We found that transmission of atypical scrapie, much more than in classical scrapie or BSE, was strongly influenced by the PrP^c^ expression levels of TgOvPrP4 inoculated mice. Whereas TgOvPrP4 mice overexpressing ∼6× the normal PrP^c^ level died after a survival periods of 400 days, those with ∼1.5× the normal PrP^c^ level died at around 700 days. The transmission of atypical scrapie in TgOvPrP4 mouse line was also strongly influenced by the *prnp* genotypes of the animal source of atypical scrapie. Isolates carrying the AF_141_RQ or AHQ alleles, associated with increased disease susceptibility in the natural host, showed a higher transmissibility in TgOvPrP4 mice. The biochemical analysis of PrP^res^ in TgOvPrP4 mouse brains showed a fully conserved pattern, compared to that in the natural host, with three distinct PrP^res^ products. Our results throw light on the transmission features of atypical scrapie and suggest that the risk of transmission is intrinsically lower than that of classical scrapie or BSE, especially in relation to the expression level of the prion protein.

## Introduction

Transmissible Spongiform Encephalopathies (TSEs) are fatal neuro-degenerative diseases that affect humans and animals, and include bovine spongiform encephalopathy (BSE) in cattle, scrapie in small ruminants, chronic wasting disease (CWD) in cervids, and Creutzfeldt-Jakob disease (CJD) in humans. The precise nature of the TSE agents is unknown, but a disease-associated (PrP^Sc^), relatively proteinase-K resistant (PrP^res^) isoform of the host cellular prion protein (PrP^c^), that co-purifies with infectivity, is supposed to be the major, if not sole, component of the infectious agent according to the “prion hypothesis” [1]–[3].

TSEs are transmissible in their species of origin, but can also cross species barriers and induce infection and/or disease after long incubation periods in other mammalian species, notably mice [4]. In this context transgenic mice expressing the prion protein of the natural host of the disease are very useful in TSEs transmission studies, as has been shown for scrapie using ovine transgenic mice [5], [6]. However, more efficient and rapid transmission was generally obtained with transgenic mice that over-expressed the physiological concentration of the prion gene whereas transgenic mice expressing physiological concentrations of PrP^c^ were less susceptible to TSE transmission and had longer incubation periods.

Scrapie transmission is highly dependent on genetic variations of the host, i.e. polymorphisms of the *prnp* ovine prion gene at codons 136 (V: valine or A: alanine), 154 (H: histidine or R: arginine) and 171 (Q: glutamine, R: arginine or H: histidine) [7], [8]. Nevertheless, the scrapie strain and, at least in *in vitro* experiments, the prion protein genotype of the animal that is the source of the infectious agent, are also important in determining scrapie transmissibility [9]–[11]. However, since 1998, a novel form of scrapie has been diagnosed [12], [13]. This disease, designated Nor98 or atypical scrapie clearly differs from classical scrapie, notably with regard to the molecular and biochemical characteristics of the corresponding PrP^res^ and to the genetic factors involved in susceptibility [14]–[16].

Transmission studies in a transgenic mouse model over-expressing high levels of the ovine PrP^VRQ^ protein had previously demonstrated transmissibility of the disease from such isolates [17], but also revealed the uniform features and similarities between cases previously described in Norway [12], then in France and Germany [13], [17]. Furthermore, regarding transmissibility in the natural host of the disease, successful experimental transmission has been reported in a single intra-cerebrally infected sheep [18] whereas no evidence of factors related to an infectious origin of the disease has been observed in the field [19].

To characterise the transmission features of the TSE agents involved in scrapie more precisely, factors determining their transmission in ovine transgenic mice (TgOvPrP4) that over-express variable individual levels of the PrP^ARQ^ ovine prion protein, were investigated. We showed that PrP^c^ expression in the brain of individual TgOvPrP4 mice could be estimated by measuring PrP^c^ levels in the sera of the corresponding mice by ELISA. The transmissibility and uniform features of the TSE agents involved in atypical scrapie were confirmed by applying this mouse model to a series of natural isolates and experimental TSE sources. However, we found that the transmissibility and survival periods of the mice were much more influenced, than in classical scrapie or BSE, by (i) the PrP^c^ expression levels of the mice and (ii) the prion protein genotypes of the ovine isolates.

## Results

We previously reported that, during transmission studies of classical scrapie and BSE, some inoculated TgOvPrP4 mice failed to accumulate detectable levels of PrP^res^ in their brains [20]. Preliminary studies with ELISA showed that the PrP^c^ levels in the brains of such mice were much lower [20] than those initially reported for PrP^c^ in the first mice bred in this mouse line [5]. We therefore decided to investigate in greater detail the individual variations of PrP^c^ expression in the TgOvPrP4 mouse line and their possible consequences on the transmission features of TSEs, notably for atypical scrapie.

### Expression levels of the ovine prion protein in TgOvPrP4 mice can be monitored by blood testing

**Figure 1:**
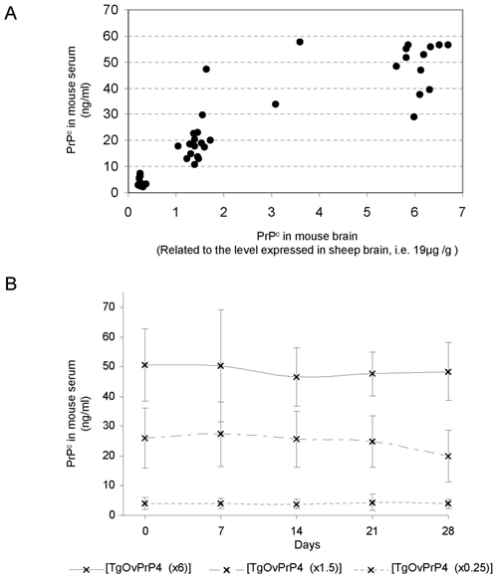
Levels of transgene expression in transgenic (TgOvPrP4) mice expressing ovine cellular prion protein (PrP^c^). (A) Quantification of seric and cerebral PrP^c^ levels by ELISA in 47 TgOvPrP4 mice. (B) Repeated measures of PrP^c^ in the serum for 4 weeks (day D0, D7, D14, D21, D28) in 47 TgOvPrP4 mice representing the 3 subpopulations with distinct PrP^c^ levels in their brain. Each line represents the average and the standard deviation of 17, 16 and 14 individual mice expressing, respectively, ∼0.25, 1.5 or 6× the PrP^c^ level expressed in a sheep brain control.

We determined the PrP^c^ expression levels in TgOvPrP4 mice by analysing the brains of a series of 47 animals produced after long-term continuous breeding of the mouse line. Results indicated the presence of three different subpopulations of TgOvPrP4 mice (17, 16 and 14 mice respectively), that expressed 5+/−1 µg/g, 27+/−3 µg/g or 109+/−20 µg/g PrP^c^ respectively, i.e. ∼0.25, 1.5 or 6× the PrP^c^ level measured in a sheep brain control (19 µg/g) (Figure 1A). In contrast, no significant individual variability was observed in 7 C57Bl/6 mice, the brains of which contained 16+/−5 µg/g of PrP^c^.

Sera from all 47 TgOvPrP4 mice were analysed to see if the three TgOvPrP4 subpopulations could be distinguished by analysing a peripheral tissue in the live animal. Before sacrifice, the serum PrP^c^ levels in the three TgOvPrP4 mouse subpopulations that respectively expressed ∼0.25, 1.5 or 6 times the PrP^c^ level expressed in a sheep brain control, were 4+/−2 ng/ml, 20+/−9 ng/ml and 48+/−10 ng/ml PrP^c^. In comparison, the sera PrP^c^ levels in 15 C57Bl/6 mice were 70+/−15 ng/ml. Repeated measures of the serum PrP^c^ levels in each TgOvPrP4 mouse prior to sacrifice, every week for 4 weeks (i.e. on day D0, D7, D14, D21 and D28), resulted in a variation coefficient (standard deviation/mean) of 16% for the 47 TgOvPrP4 mice and demonstrated the reliability of the discrimination of the three TgOvPrP4 subpopulations (Figure 1B). Statistical analyses confirmed that the serum PrP^c^ levels in [TgOvPrP4 (x0.25)] were significantly lower than those in [TgOvPrP4 (x1.5)] or [TgOvPrP4 (x6)] (t-test, p<0.0001 and p<0.0001 respectively) and that the serum PrP^c^ levels in [TgOvPrP4 (x1.5)] were significantly lower than those in [TgOvPrP4 (x6)] (t-test, p<0.0001). This implied that PrP^c^ expression in the brains of TgOvPrP4 mice could be monitored by measuring the PrP^c^ levels in the mouse sera. Thus serum PrP^c^ levels <10 ng/ml, 10–40 ng/ml or >40 ng/ml respectively were found to identify TgOvPrP4 mice expressing ∼0.25, 1.5 or 6× the PrP^c^ level measured in a sheep brain control.

### Atypical scrapie is transmissible in TgOvPrP4 ovine transgenic mice

Twelve brain homogenates from atypical scrapie natural isolates were intracerebrally inoculated into the TgOvPrP4 mouse line (Table 1). These isolates included 7 sheep and 1 goat with *prnp* alleles associated with increased susceptibility to atypical scrapie (AF_141_RQ or AHQ) (group 1a) and 4 sheep without such alleles (group 1b). Most of the inoculated mice (85/90 in group 1a and 37/41 in group 1b) developed clinical symptoms, including hunched posture, weight loss, reduced mobility, tail chewing, clasping and difficulty or impossibility to stand up. In 11 of the 12 experiments, the first deaths occurred between 350–450 days post-inoculation (d.p.i.). Western blot (detecting PrP^res^) or IHC (detecting PrP^sc^) analyses indicated the presence of PrP^res^/PrP^sc^ in 87% and 82% of the mice inoculated with group 1a and group 1b isolates respectively.

**Table 1:** Natural scrapie isolates transmission results to TgOvPrP4 ovine transgenic mice.

Inoculum (1)	Genotype (2)	Survival periods (dpi) (mean±SD (range))	No. of PrPres or PrPsc positive animals/no. Inoculated
Group 1a			
Atyp 1	**AF_141_RQ**/**AF_141_RQ**	386±13 (372–408)	11/11
Atyp 2(α)	**AF_141_RQ**/AL_141_RR	401±42 (342–477)	8/9
Atyp 3	**AF_141_RQ**/AL_141_RR	443±112 (342–653)	8/10
Atyp 4	**AF_141_RQ**/AL_141_RR	454±119 (341–650)	11/12
Atyp 10(β)	**AF_141_RQ**/AL_141_RR	596±117 (446–757)	8/9
Atyp 8	**AF_141_RQ**/AL_141_RQ	517±137 (379–684)	11/12
Atyp 5	**AL_141_HQ**/AL_141_RR	520±154 (384–740)	9/10
Atyp 12(Goat)	**AL_141_HQ**/**AL_141_HQ**	601±143 (407–769)	8/12
Group 1b			
Atyp 9	AL_141_RR/AL_141_RR	596±176 (406–817)	8/10
Atyp 7	AL_141_RR/AL_141_RR	540±196 (349–796)	9/9
Atyp 6	AL_141_RR/AL_141_RR	492±108 (393–700)	8/9
Atyp 11	AL_141_RH/AL_141_RH	623±86 (518–743)	8/12
Group 2			
Class^usual^ 1	ARQ/ARQ	314±57 (259–477)	10/11
Class^usual^ 2(β)	ARQ/ARR	640±62 (559–775)	12/12
Class^usual^ 3(α)	ARQ/VRQ	666±101 (496–876)	7/12
Group 3			
Class^CH1641-like^ 1	VRQ/VRQ	248±50 (187–357)	10/10
Class^CH1641-like^ 2	ARQ/ARQ	257±57 (202–369)	7/7
Class^CH1641-like^ 3	ARQ/ARQ	240±61 (184–419)	12/12
Class^CH1641-like^ 4	VRQ/VRQ	364±61 (230–408)	12/12
Class^CH1641-like^ 5	ARQ/ARQ	235±53 (142–331)	8/8
Class^CH1641-like^ 7	ARQ/ARQ	235±26 (197–274)	8/8
Class^CH1641-like^ 8	ARQ/ARQ	240±21 (218–262)	7/8

(1) Animals were inoculated intracerebrally with 2 mg brain tissue.

(2) Genotype of sheep or goat (amino acids 136, 141, 154, and 171).

(Goat) Goat isolate.

(α) Isolates detected in the flock α.

(β) Isolates detected in the flock β.

Natural inoculums were divided into 4 different groups, all originating from active surveillance of TSEs in small ruminants. Group 1a: atypical scrapie cases with *prnp* alleles associated with increased susceptibility to atypical scrapie (AF_141_RQ or AHQ). Group 1b: atypical scrapie cases without *prnp* alleles associated with increased susceptibility to atypical scrapie. Group 2: classical usual scrapie cases. Group 3: CH1641-like scrapie cases. Detection of PrP^res^/PrP^sc^ was performed by Western blot or IHC analyses.

For comparison, TgOvPrP4 mice were intracerebrally inoculated with 10 natural scrapie isolates, three classical scrapie with usual molecular features (i.e. high molecular weight of PrP^res^ compared to BSE or CH1641 experimental scrapie source) (group 2) and seven “CH1641-like” scrapie isolates (group 3) (Table 1). Eighty-six of the 99 inoculated mice developed clinical symptoms similar to those previously described in mice inoculated with atypical scrapie isolates. Western blot (detecting PrP^res^) or IHC (detecting PrP^sc^) analyses revealed PrP^res^/PrP^sc^ in 93% of the animals.

Two of the three classical scrapie isolates with usual molecular features (Class^usual^ 2 and Class^usual^ 3) were identified in flocks (β and α respectively) from which a case of atypical scrapie (Atyp 10 and Atyp 2 respectively, both belonging to group 1a), had been identified.

These two classical scrapie cases showed long survival periods (640 and 666 d.p.i. respectively) compared to other classical scrapie in TgOvPrP4 mice [5], [21].

Isolates from the two pairs of atypical and classical scrapie cases originating from the same flock were also inoculated into C57Bl/6 wild-type mice (Table 2). The 2 classical scrapie isolates (Class^usual^ 2 and 3) transmitted the disease to these mice (mean survival periods of 531 and 584 d.p.i) and produced 17% and 35% of PrP^res^/PrP^sc^ positive mice, respectively. Although the survival range and mean survival periods (532 and 607 d.p.i. respectively) were comparable, PrP^res^/PrP^sc^ were not detected in any of the C57Bl/6 mice inoculated with the 2 atypical scrapie isolates.

**Table 2:** Natural scrapie isolates transmission results to C57Bl/6 wild-type mice.

Inoculum(1)	Genotype(2)	Survival periods (dpi) (mean±SD (range))	No. of PrPres or PrPsc positive animals/no. Inoculated
Atyp 2(α)	**AF_141_RQ**/AL_141_RR	532±118 (285–685)	0/17
Atyp 10(β)	**AF_141_RQ**/AL_141_RR	607±107 (369–860)	0/18
Class^usual^ 2(β)	ARQ/ARR	531±94 (317–645)	3/18
Class^usual^ 3(α)	ARQ/VRQ	584±52 (481–642)	6/17

(1) Animals were inoculated intracerebrally with 2 mg brain tissue.

(2) Genotype of sheep or goat (amino acids 136, 141, 154, and 171).

(α) Isolates detected in the flock α.

(β) Isolates detected in the flock β.

Detection of PrP^res^/PrP^sc^ was performed by Western blot or IHC analyses.

Finally TgOvPrP4 mice were also intracerebrally inoculated with 6 experimental TSE sources, three experimental isolates from sheep (group 4), and three experimental strains initially derived from wild-type C57Bl/6 mice (group 5) (Table 3). Sixty-five of the 70 inoculated mice developed clinical symptoms and Western blot or IHC analyses revealed PrP^res^/PrP^sc^ in 96% of the animals.

**Table 3:** Experimental TSEs transmission results to TgOvPrP4 ovine transgenic mice.

Inoculum(1)	Genotype(2)	Survival periods (dpi) (mean±SD (range))	No. of PrPres or PrPsc positive animals/no. Inoculated
Group 4			
SSBP1	VRQ/VRQ	437±107 (330–656)	11/12
CH1641	AXQ/AXQ	231±49 (186–355)	12/12
BSE^OVINE^	ARQ/VRQ	436±36 (357–461)	10/10
Group 5			
BSE	–	375±51 (341–531)	12/12
C506M3	–	332±25 (295–377)	12/12
87V	–	290±68 (211–390)	10/12

(1) Animals were inoculated intracerebrally with 2 mg brain tissue.

(2) Genotype of sheep or goat (amino acids 136, 141, 154, and 171).

Experimental inoculums were divided into 2 different groups. Group 3: small ruminants intracerebrally inoculated with various TSEs. Group 4: experimental strains initially derived from wild-type mice. Detection of PrP^res^/PrP^sc^ was performed by Western blot or IHC analyses.

### Transmission efficacy of atypical scrapie is at least partly conditioned by the levels of ovine PrP^c^ in mice

**Figure 2:**
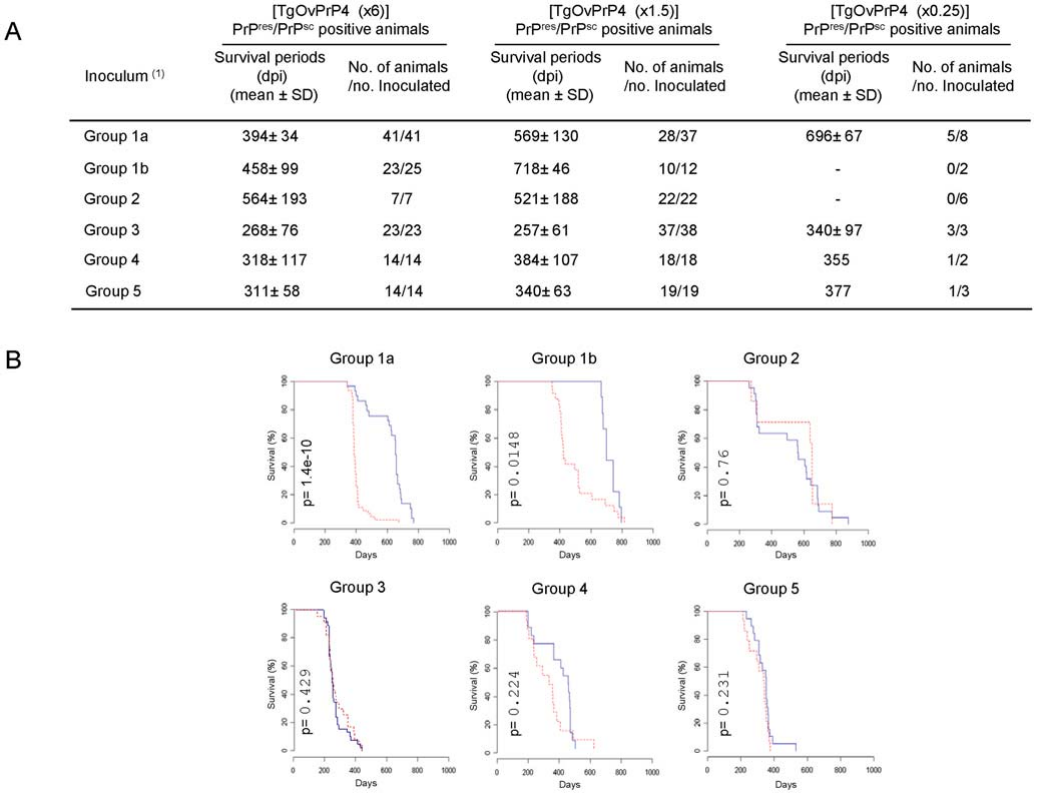
Effects of variable PrP^c^ expression levels in TgOvPrP4 mice on TSEs transmission. Survival periods of PrP^sc^ positive TgOvPrP4 mice are shown according the level of PrP^c^ expression (∼0.25, 1.5 or 6× the PrP^c^ level expressed in a sheep brain control) and to the inoculums; Group 1a: atypical scrapie isolates carrying the AF_141_RQ or AHQ alleles, Group 1b: atypical scrapie isolates without the AF_141_RQ or AHQ alleles, Group 2: usual classical scrapie isolates, Group 3: CH1641-like scrapie isolates, Group 4: experimental isolates (SSBP1, CH1641, BSE^OVINE^), Group 5: mouse-adapted strains (BSE, C506M3, 87V). (A) Transmission results. (B) Kaplan-Meier survival curves of PrP^res^/PrP^sc^ positive mice expressing ∼1.5 (blue line) or ∼6× (red line) the PrP^c^ level expressed in a sheep brain control for each of the 6 different groups. p-values represent log-rank differences in cumulative survival between mice expressing ∼1.5 and ∼6× the PrP^c^ level expressed in a sheep brain control.

Transmission results in TgOvPrP4 mice were further analysed in relation to the three patterns of ovine PrP^c^ expression in mice, by analysing serum sampled from each mouse at the time of inoculation (Figure 2).

For animals inoculated with classical scrapie isolates (groups 2 and 3) or experimental TSE sources (groups 4 and 5), negative PrP^res^/PrP^sc^ brains have been observed, but only in [TgOvPrP4 (x0.25)] mice (9/14) or very rarely in [TgOvPrP4 (x1.5)] mice (1/97), whereas all the 58 mice from the other group were PrP^res^/PrP^sc^ positive (Figure 2A). In contrast, some PrP^res^/PrP^sc^ negative mice were found in all 3 TgOvPrP4 subpopulations inoculated with atypical scrapie (group 1a and group 1b), i.e. not only in [TgOvPrP4 (x0.25)] (5/10), but also in a significant proportion of [TgOvPrP4 (x1.5)] (11/49) and even in [TgOvPrP4 (x6)] (2/66) mice.

Differences between the survival periods of PrP^res^/PrP^sc^ positive mice in the three TgOvPrP4 subpopulations were only observed for the two atypical scrapie groups. The differences in mean survival periods between [TgOvPrP4 (x1.5)] and [TgOvPrP4 (x6)] were 43, 11, 66 or 29 days for classical scrapie or BSE (groups 2, 3, 4 and 5 respectively), but were 175 and 260 days for atypical scrapie (groups 1a and 1b respectively). These survival figures for PrP^res^/PrP^sc^ positive mice (Figure 2B) were subjected to statistical analyses using the Log-Rank test and the Cox proportional-hazard regression models. The Log-Rank test revealed that the differences in survival between [TgOvPrP4 (x1.5)] and [TgOvPrP4 (x6)] mice inoculated with atypical scrapie were significant (Log-Rank, p<0.0001 and p = 0.015 in groups 1a and 1b respectively) whereas no significant differences were found between the other groups (Log-Rank, p = 0.76, p = 0.429, p = 0.224, p = 0.231 for groups 2, 3, 4 and 5 respectively). These results were consistent with the Cox proportional-hazard regression model results which showed a significant effect of serum PrP^c^ levels on the survival period for mice inoculated with atypical scrapie (Wald test p = <0.0001 for both groups 1a and 1b), but did not suggest any effect of PrP^c^ expression levels in the PrP^res^/PrP^sc^ positive mice inoculated with classical scrapie or BSE (Wald test, p = 0.51, p = 0.09, p = 0.053 and p = 0.14 in groups 2, 3, 4 and 5 respectively).

The hazard ratios associated with the serum PrP^c^ levels for atypical scrapie are indicated in Table 4. The PrP^c^ expression levels significantly increased the risk of death whatever the genotype group (groups 1a, 1b); an increase of one unit (ng/ml) in the serum PrP^c^ level corresponded to an increased risk of death of 3%. When the serum PrP^c^ level of 4 ng/ml ([TgOvPrP4 (x0.25)] mean value) was set as the baseline category, the hazard ratios and their 95% confidence intervals were 1.53 (95% CI: 1.34–1.76) for a serum PrP^c^ level of 20 ng/ml ([TgOvPrP4 (x1.5)] mean value) and 3.24 (95% CI: 2.22–4.71) for a serum PrP^c^ level of 48 ng/ml ([TgOvPrP4 (x6)] mean value); this corresponded to an increased risk of 53% and 224% for the [TgOvPrP4 (x1.5)] and [TgOvPrP4 (x6)] groups respectively compared to the [TgOvPrP4 (x0.25)] group.

**Table 4:** Statistical analysis of PrP^c^ levels influencing survival period of TgOvPrP4 mice inoculated with atypical scrapie.

PrP^c^ level	Hazard ratio	95% CI
48 ng/ml: [TgOvPrP4 (x6)]	1*	–
20 ng/ml: [TgOvPrP4 (x1.5)]	1.53	(1.34–1.76)
48 ng/ml: [TgOvPrP4 (x6)]	3.24	(2.22–4.71)

* PrP^c^ level set as the baseline category.

Hazard ratios calculated from model used to identify predictors of survival for mice inoculated with atypical scrapie isolates and illustrating the effect associated with PrP^c^ sera levels (the mean of each subpopulation were chosen to illustrate the effect of the PrP^c^ sera level).

### Transmission of atypical scrapie is conditioned by the *prnp* genotype of sheep at codons 141 and 154

The transmission results for atypical scrapie were also analysed in relation to the *prnp* genotype of the sheep that were the source of the infectious agent, by comparing group 1a (animals with increased susceptibility to atypical scrapie and with the AF_141_RQ or AHQ *prnp* alleles) and group 1b (animals without AF_141_RQ or AHQ *prnp* alleles) [14], [15].

The Kaplan-Meier survival curves for PrP^res^/PrP^sc^ positive mice (Figure 2B) showed that 98% of the [TgOvPrP4 (x6)] mice in group 1a died at around 400 d.p.i and 25% of the [TgOvPrP4 (x1.5)] mice died before 600 d.p.i. In contrast, in group 1b, 20% of the [TgOvPrP4 (x6)] mice died after 600 d.p.i and all the [TgOvPrP4 (x1.5)] mice died after 666 d.p.i.. The significant difference in survival between groups 1a and 1b, according to the Cox proportional-hazard regression model was confirmed by statistical analyses (Wald test p<0.0001). For the hazard ratios, the risk was 4.32 fold (95 CI: 2.5–7.19) higher in group 1a than in group 1b whatever the PrP^c^ expression level (Table 5). The risk was thus significantly higher in group 1a than in group 1b.

**Table 5:** Statistical analysis of *prnp* genotype influencing survival period of TgOvPrP4 mice inoculated with atypical scrapie.

Category	Hazard ratio	95% CI
Group 1b	1**	–
Group 1a	4.32	(2.50–7.19)

** Group set as the baseline category.

### A consistent biochemical signature of atypical scrapie in TgOvPrP4 mice

**Figure 3:**
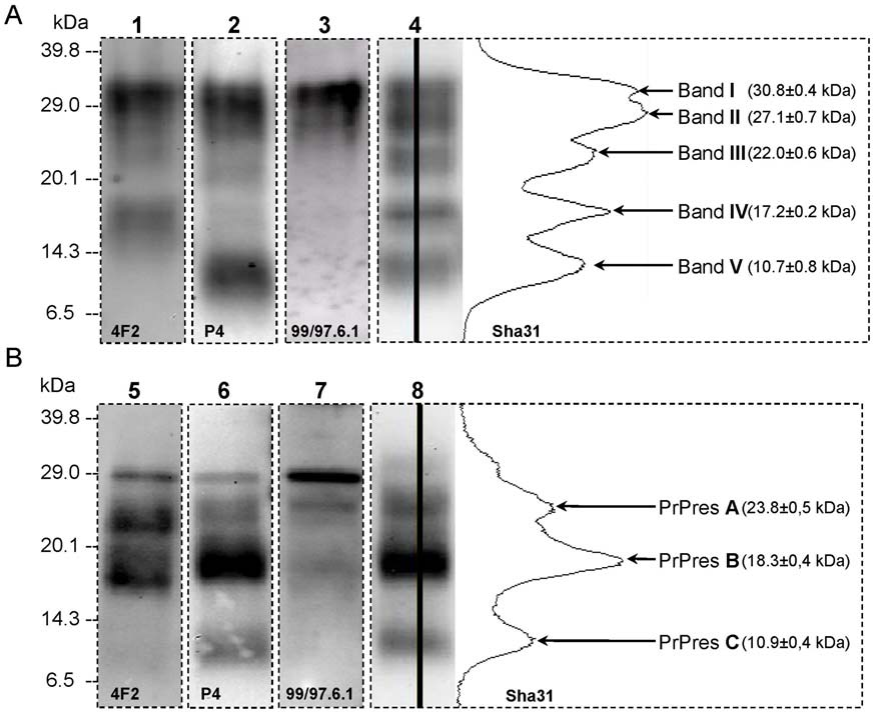
Western blot profiles of PrP^res^ in TgOvPrP4 mice infected with atypical scrapie. Results are shown before (A) and after (B) PNGase deglycosylation using monoclonal antibodies 4F2 (lanes 1, 5), P4 (lanes 2, 6) (N-terminal), Sha 31 (lanes 4, 8)(core) and 99/97.6.1 (lanes 3, 7)(C-terminal). Western blot profiles with Sha 31 antibody are shown with the curve of chemiluminescence measured along the lanes. Apparent molecular weights were means from 30 mice before PNGase deglycosylation and from 7 mice after PNGase deglycosylation.

Finally, detailed biochemical studies were performed to characterize the PrP^res^ from mouse brains. When the Sha 31 monoclonal antibody was used for PrP^res^ detection, the usual 3-band pattern (between 18–20 and 30 kDa), that became a single band pattern (18–20 kDa) after PNGase deglycosylation, was observed in all TgOvPrP4 and C57Bl/6 PrP^res^ positive mice inoculated with classical scrapie or BSE (groups 2, 3, 4 and 5) (data not shown). In contrast, a complex pattern with five major bands between 31 and 10 kDa (named bands I to V), was observed in all 53 PrP^res^ positive TgOvPrP4 mice inoculated with the 12 atypical scrapie cases (Figure 3A). Three PrP^res^ fragments with apparent molecular masses of ∼24, 18 and 11 kDa (PrP^res^ A, B and C respectively) were detected after PNGase deglycosylation (Figure 3B).

**Figure 4:**
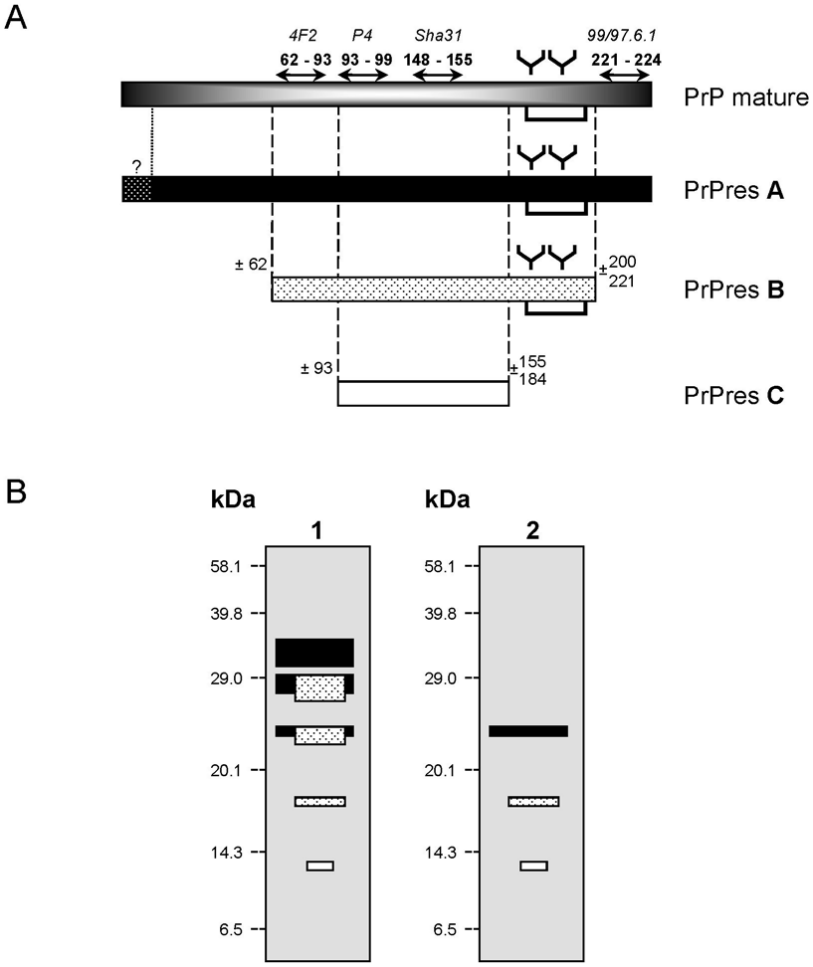
Schematic representation of PrP^res^ fragments identified in atypical scrapie. (A) Diagram illustrating PrP^res^ A, B and C sequences in atypical scrapie, with location of epitopes recognised by monoclonal antibodies used during the study and estimated cleavages of PrP^res^ fragments. (B) Diagram of Western blot profiles of PrP^res^ detected using Sha 31 monoclonal antibody before (1) and after (2) PNGase deglycosylation.

The biochemical features of PrP^res^ in mice inoculated with atypical scrapie, before and after PNGase deglycosylation, were compared by using different monoclonal antibodies that recognised different prion protein sequences along the protein, including 4F2 (62–93), P4 (93–99), Sha 31 (148–155) and 99/97.6.1 (221–224). After PNGase deglycosylation, we noted non-specific immunostaining at ∼29 kDa with all monoclonal antibodies (4F2, P4 and 99/97.6.1) revealed with peroxidase-labeled conjugates against mouse immunoglobulins, also present in uninfected mice. All three different PrP^res^ protease cleavage products were detected by antibodies P4 and Sha 31, whereas 4F2 did not detect PrP^res^ C (Figure 3B). The 99/97.6.1 antibody showed lower sensitivity but reacted more strongly with PrP^res^ A than with PrP^res^ B. Prior to deglycosylation, 99/97.6.1 did not immunostain bands III, IV and V (21, 17 and 11 kDa) (Figure 3A). All together our data suggest that the C-terminal epitope recognised by this latter antibody might be present in PrP^res^ A but only faintly in PrP^res^ B fragments (Figure 4).

PrP^res^ Western blot profiles from 30 mice representative of the different sheep genotypes and of the three subpopulations of TgOvPrP4 mice were subjected to repeated analyses and quantification. The variability of the apparent band molecular weights between mice was only slight with a coefficient variation (standard deviation/mean) of 2% for bands I to IV and 7% for band V (Figure 3A), and similar to that observed for repeated measures from the same brain sample (2.1+/−1.2% for bands above 16 kDa and 4.0+/−1.7% for the band around 11 kDa). The variability was similar following PNGase deglycosylation of PrP^res^ in 7 mice from three different experiments (Atyp 1, 2, 6), with a coefficient variation of 2% for PrP^res^ A and B and 4% for PrP^res^ C (Figure 3B). These data do not indicate any change in PrP^res^ patterns associated with either the PrP^c^ levels of the mice or the *prnp* genotypes of the sheep or goats.

## Discussion

Characterization of the infectious agents involved in TSEs has historically required transmission in inbred wild-type mice, such as C57Bl, VM or RIII [4], [22], [23]. However some TSEs, such as the CH1641 experimental scrapie source [24] or some human TSEs [25] fail to transmit to wild-type mice of any genotype. The recent availability of transgenic mice expressing the prion gene of the natural host of the disease has facilitated TSE transmission studies, as in the use of ovine transgenic mice for scrapie or BSE [5], [20], [21]. The strain-specific molecular diversity of the classical scrapie or BSE sources was faithfully reproduced in the TgOvPrP4 ovine transgenic mouse model that we have developed. However, a novel TSE, called atypical scrapie or Nor98, has recently been identified in sheep and goats throughout Europe [12]. In several countries this disease is now more frequent than classical scrapie and in some countries atypical scrapie has been recognized in the absence of classical scrapie [26]–[28]. The experimental transmissibility of this disease was established in a transgenic mouse model (tg338) that strongly over-expresses (8- to 10-fold) the VRQ allele of ovine prion protein, from a series of 10 French atypical scrapie cases and 2 Norwegian Nor98 isolates [17]. In contrast, experimental transmission was not achieved in wild-type mice [12], [17] or in bank voles [29]. Overall this clearly confirmed that atypical scrapie is a genuine TSE associated with infectious prions.

In this study we confirmed the transmissibility of atypical scrapie, after intracerebral challenge, in another ovine transgenic mouse model (TgOvPrP4), but failed to transmit the disease from 2 cases into wild-type mice. Twelve cases, including the 10 French isolates previously transmitted to the tg338 mouse line [17], were successfully transmitted to TgOvPrP4 mice that over-expressed the AL_141_RQ prion protein. Although this *prnp* genotype of TgOvPrP4 mice is only rarely identified in sheep with atypical scrapie [13]–[16], [26], [30]–[33], most of the inoculated mice accumulated the pathological prion protein in their brain. Biochemical analysis of PrP^res^ showed a fully conserved pattern comparable to that previously described in the natural host [15], i.e. a similar complex pattern including 5 major bands derived from 3 distinct PrP^res^ products. Both PrP^res^ fragments cleaved at both N- and C-terminal ends, and the uncleaved (or marginally cleaved) PrP^res^, were consistently maintained after transmission into ovine transgenic mice (Figure 4). The consistency of the Western blot profiles, whatever the isolate, and in line with previous studies in tg338 ovine transgenic mice, does not suggest any diversity in atypical scrapie. This consistent PrP^res^ glycoprofile is somewhat reminiscent of certain PrP^res^ features in patients with Gerstmann-Straüssler-Scheinker syndrome [34]–[36].

This study enabled us to extend the descriptive profile of our TgOvPrP4 mouse model and examine TSE transmission factors in relation to the level of expression of the prion protein by the infected host. After long-term breeding of the mouse line we indeed found three TgOvPrP4 mice subpopulations expressing in their brain ∼0.25, 1.5 or 6× the PrP^c^ level measured in a sheep brain control. We then decided to evaluate the possible influence of these differences in PrP^c^ levels in the central nervous system on TSE development, following the inoculation of 3 mouse-adapted strains (BSE, C506M3, 87V), 3 experimental small ruminants isolates (SSBP1, CH1641, BSE^OVINE^) and 22 small ruminant natural isolates collected by active surveillance of TSEs in sheep and goats (10 classical scrapie isolates, including 3 usual isolates as shown by PrP^res^ molecular analysis and 7 “CH1641-like” isolates; 12 atypical scrapie isolates). This panel of samples thus represents the molecular diversity of TSEs in small ruminants, especially with the three basic PrP^res^ phenotypes possibly demonstrated by Western blot in natural scrapie. Strikingly the influence of the PrP^c^ expression level was much more obvious for the atypical scrapie isolates, and a considerable delay in disease onset was associated with a decrease in PrP^c^ expression levels,. TgOvPrP4 mice with higher PrP^c^ levels (∼6×) died after shorter periods (around 400 days) than mice with lower PrP^c^ levels (∼1.5×) (around 700 days). Comparisons of different transgenic mouse lines already indicated that the expression levels of the prion protein were inversely related to the incubation period in classical scrapie [6]. Nevertheless, to the best of our knowledge, the variable influence of PrP^c^ expression levels according to the form of prion disease defined by its essential molecular features, has never been described and is here reported between different animals in a same transgenic mouse line. However, although the development of atypical scrapie appears to be much more facilitated by increased PrP^c^ expression, this also suggests that the risk of transmission in animals expressing physiological levels of the protein would be relatively low. This characteristic, in addition to the species barrier, would help to explain the failure to transmit atypical scrapie to wild-type mice (RIII, VM and C56Bl mice) [17] or bank voles [29], unlike classical scrapie. Besides, a major influence of the PrP^c^ expression levels has been demonstrated in transgenic mice over-expressing (i) mutated human prion protein linked to Gerstmann–Sträussler-Scheinker syndrome [37], [38] or (ii) a prion protein with a nine-octapeptide insertion associated with a human familial prion disease [39], [40]. In both cases disease development was strongly conditioned by the levels of transgene expressions, not only following inoculations of brain tissues from affected patients, but also spontaneously.

Our demonstration that atypical scrapie was much more influenced by PrP^c^ expression levels than classical scrapie or BSE was possible in this mouse model solely because our approach was based on serum PrP^c^ measurements. These were indeed found to reflect the levels of PrP^c^ expression in the brain. In this model protein expression was controlled by the neuron-specific enolase promoter and neither RT-PCR or Western blot analyses were able to detect expression of the *prnp* gene in non nervous tissues [5], [41]. One explanation of the close relationship between serum and cerebral PrP^c^ might be related to a release of PrP^c^ from the central nervous system into the blood, as was also shown for some other neuronal proteins [42]. It is noteworthy that the plasma concentration of PrP^c^ in wild-type C57Bl mice (∼70+/−15 ng/ml) is much higher than in TgOvPrP4 mice, even in [TgOvPrP4 (x6)] with the highest level of expression (48+/−10 ng/ml). This suggests that a fraction of the PrP^c^ in the serum of wild-type mice could originate from the nervous system, under physiological conditions. Importantly, direct evidence that PrP^c^ readily crosses the blood brain barrier in both brain-to-blood and blood-to-brain directions has recently been reported [43]. Regarding the TgOvPrP4 mouse line, the presence of [TgOvPrP4 (x0.25)] animals which express low levels of PrP^c^ clearly represents a drawback for transmission studies of TSEs. This can however be circumvented by assessing the PrP^c^ expression levels in each individual mouse prior to its use in animal experiments. Furthermore, although this subpopulation increased following long-term random breeding of the mouse line, blood testing is also used to monitor PrP^c^ expression levels during breeding in order to eliminate those breeders that produce progeny with a high proportion of poorly expressing animals. It should be noted that no indication of variation of transgene expression was found in a similarly produced mouse line (TgOvPrP59) originating from different founders [44], [45], which makes it unlikely that our observations in the TgOvPrP4 mouse line result from factors intrinsic to the transgene construct and/or the genetic background of the mice. However, the loss of transgene expression in a transgenic line is not unprecedented and two types of explanation are generally provided to account for such a loss. These include (1) integration of the transgene in an heterochromatin-rich region which results in silencing of the transgene in some cells (position effect variegation) [46] or (2) the repetitive nature of the transgene arrays which induces the formation of heterochromatin at the sites of integration and leads to gene silencing [47]. In addition, our genetic construction does not contain any insulators, which suggests that heterochromatin propagation might be a cause of transgene extinction in some animals [48].

Concerning the factors involved in the transmission of atypical scrapie, we were also surprised to demonstrate the influence of the *prnp* genotypes of the sheep or goat donors on development of the disease in TgOvPrP4 inoculated mice. This had not been observed in tg338 mice, which overall showed more rapid incubation of the disease [17]. Lower PrP^c^ expression levels in TgOvPrP4 mice were required to transmit atypical scrapie, when the isolates carried the AF_141_RQ or AHQ alleles associated with increased susceptibility to the disease in sheep [14]. The same analysis of possible differences associated with the susceptible or resistant genotype, as here reported in atypical scrapie, could not be done for classical scrapie or BSE considering the available isolates. Among these sources only one classical scrapie isolates (Class^usual^ 2) carried the ARR allele known to be associated with resistance to clinical scrapie [49], [50]. Although the ARR allele is rarely observed in classical scrapie we here confirmed, by both PrP^res^ analyses in sheep and bioassays in wild-type and ovine transgenic mice, that it is possible to detect classical scrapie in relatively resistant animals during the implementation of flock analysis of scrapie cases [51]. Overall, given the limited number of samples available we cannot exclude a possible influence of the *prnp* genotypes for classical scrapie or BSE. The results observed with atypical scrapie are difficult to explain and we cannot exclude differences in infectious titres between the two groups of samples examined, in relation to (i) possible differences in the distribution of infectivity within the CNS [52] and/or (ii) different stages in the incubation periods in sheep and goats. On the other hand the AF_141_RQ or AHQ *prnp* alleles were associated with a lower conformational stability of the ovine PrP^c^ protein [53], which might be associated with an increased capacity of the PrP^sc^ of such genotypes to transconform PrP^c^. It is noteworthy that only one successful experimental transmission of atypical scrapie has so far been reported in sheep, which was obtained in an AHQ homozygous sheep intracerebrally inoculated with an atypical scrapie isolate from an AHQ homozygous sheep [18]. However the apparently spontaneous occurrence of three cases of atypical scrapie in genetically susceptible sheep was also reported in a flock derived from sheep imported from New Zealand, a country regarded as scrapie-free [54].

Based on our findings, the possibility that variations in the expression of ovine PrP^c^ might be factors involved in susceptibility to atypical scrapie in sheep and goats, is open to question. A second proposal could question whether a failure of the protein quality-control systems might also be involved in susceptibility to atypical scrapie. Recent studies have provided new evidence for a role of these systems in the conversion of PrP^c^ to an abnormal form of prion protein, with either impairment of proteasomal degradation or endoplasmic reticulum stress, leading to the generation of a misfolded form of prion protein [55]–[57]. Transgenic mice were also reported to develop a spontaneous neurological disease in the absence of PrP^sc^ when preferentially expressing a transmembrane form of prion protein (PrP^Ctm^) [58], [59], which would imply that ^Ctm^PrP is a key component in the pathway of neurodegeneration as in Gerstmann–Sträussler-Scheinker syndrome.

However our data do emphasize the unresolved questions regarding the origin of atypical scrapie, which as yet remains unknown. A spontaneous origin of the disease is suspected, and importantly, no evidence of natural transmissibility in small ruminants has been observed [33], [60]. The results of our novel approach, based on monitoring prion protein expression in animals from the same transgenic mouse line, suggest that the risk of transmission of atypical scrapie is intrinsically lower than that of classical scrapie or BSE, especially in relation to the expression level of the prion protein.

## Materials and Methods

### Biological samples

The samples differed by their origin and nature (natural scrapie isolates and experimental samples). Groups 1a, 1b, 2 and 3 are natural samples collected by active surveillance of scrapie, suggesting that these most probably have been sampled before any clinical disease. In contrast, groups 4–5 included experimental samples derived from animals intracerebrally inoculated with TSE then sacrificed at the stage of clinical disease.

Natural scrapie isolates (Table 1) were obtained from 21 sheep and 1 goat previously classified as atypical (group 1a and 1b: categorized according to the presence/absence of *prnp* alleles associated with increased susceptibility to this disease (AF_141_RQ or AHQ)) (n = 12, Atyp), classical usual (group 2) (n = 3, Class^usual^) or “CH1641-like” (n = 7, Class^CH1641-like^) scrapie cases according to the PrP^res^ molecular phenotype of the isolates. Group 2 contains classical scrapie isolates with “usual” PrP^res^ molecular features, i.e. a higher apparent molecular mass compared to ovine BSE or to the CH1641 experimental scrapie isolate [61], whereas group 3 contains unusual “CH1641-like” isolates that show similar PrPres molecular features to that of CH1641 [62]–[64]. Natural scrapie isolates included two pairs of atypical/classical usual scrapie cases originating from the same flocks (Atyp 2/Class^usual^ 3 in flock α and Atyp 10/Class^usual^ 2 in flock β). The genotypes and PrP^res^ biochemical features of atypical cases have been described previously [15].

Experimental isolates (group 4) (SSBP/1, CH1641, BSE ^OVINE^) were obtained from small ruminants intracerebrally inoculated with SSBP/1 (Sheep Scrapie Brain Pool 1) and CH1641 scrapie isolates (kindly provided by N. Hunter, Institute for Animal Health, Edinburgh, United Kingdom) or with a French bovine BSE case [61]. Transmission studies of these isolates in TgOvPrP4 and C57Bl/6 mice have been reported previously [20].

Experimental strains (group 5) (BSE, C506M3, 87V), derived from mouse-adapted strains [20], were used at a second passage from TgOvPrP4 mice.

### Ovine PrP^c^ levels in mouse sera and brain

The TgOvPrP4 ovine transgenic mice have already been described [5]. These mice express the ovine prion protein (A136 R154 Q171) open reading frame that had been inserted into the pNSE-Ex4 vector (neuron specific enolase promoter). Transgenic founders were crossed with prion protein knockout mice to obtain homozygous mice for both the ovine *prnp* transgene and the deletion of the murine *prnp* locus.

The levels of PrP^c^ in TgOvPrP4 and C57Bl/6 (Charles River, L'Arbresle, France) mouse serum or brain were measured using an ELISA (Enzyme Linked Immuno Sorbent Assay) method as previously described [65], [66], involving two different two-site immunometric assay based on two different combinations of monoclonal antibodies. The capture antibodies were 11C6 (recognised epitope unidentified) [67] and SAF34 (octa-repeat region) [68] for mouse sera and brains respectively. In each case, the tracer antibody was Bar 224 (141–152 prion protein sequence in human) as Fab' acetylcholinesterase conjugate. Sera samples were centrifuged (5000 rpm, 5 min) prior to dilution of the sera (1/5 for TgOvPrP4, 1/10 for C57Bl/6) in EIA buffer (100 mM potassium phosphate buffer pH 7.4, 0.15M NaCI, 0.1% sodium azide). Brain samples were prepared as 20% homogenates in 5% (w/v) glucose prior to dilution (1/100) in EIA buffer. Samples (100 µl per well in duplicate) were loaded in ELISA microtiter plates (Immunoplate, Maxisorp, Nunc) coated with capture antibodies. After two hours at room temperature, the plates were washed with EIA buffer before addition of the conjugate antibody (2 Ellman's units per ml EIA buffer). After 2 hours at room temperature, the plates were washed again in potassium phosphate buffer 10 mM, and Tween 20 (0.05%, pH 7.4). The colorimetric substrate (Ellman's reagent) was then added (200 µl per well). Absorbance was measured at 410 nm after 30 min of enzyme reaction. The concentration of PrP^c^ was estimated by reference to a standard curve established with a series dilutions of sheep recombinant prion protein purified from *E coli* [69]. This gave a reproducible linear curve with a detection limit close to 2 ng/ml for serum and 2 µg/g for brain.

### Mouse transmission assays

Prior to inoculation, a sample of serum was taken from each TgOvPrP4 mouse. Experimental groups of four to six week old female TgOvPrP4 or C57Bl/6 (Charles River, L'Arbresle, France) mice were anaesthetized (80 µl of 0.8% ketamin –0.12% xylazine) then inoculated intracerebrally (20 µl per mouse) with a 10% solution (wt/vol) of homogenates prepared from CNS samples of TSE cases in glucose (5%). The mice were cared for and housed in the Biohazard prevention area (A3) of our institution, according to the European guidelines (directive 86/609/EEC) and the French Ethical Committee (decree 87–848), and were supplied with food and drink *ad libitum*. They were checked at least twice weekly for the presence of clinical signs. When signs occurred, the mice were monitored daily and were sacrificed with an anaesthetic solution overdose (200 µl of 0.8% ketamin –0.12% xylazin) if they exhibited any signs of distress or confirmed evolution of clinical signs of prion disease. A few animals were found dead. The whole brain of every second mouse was frozen and stored at −80°C before Western blot analysis for PrP^res^ detection ; the other brains were fixed in 10% buffered formalin solution for PrP^sc^ histopathological analysis. All procedures were agreed (N°98) by the CREEA (Regional Committee for Ethical Experimentation on Animals).

### Statistical analysis

Plots of survival curves obtained by Kaplan-Meier method were displayed for TgOvPrP4 mice overexpressing 1.5 or 6× the PrP^ARQ^ ovine prion protein in each of the six inoculum groups. Log-Rank tests were computed for each group to assess any survival difference between the two levels. Cox proportional-hazard regression models were used to identify predictors of the survival of PrP^res^/PrP^sc^ positive inoculated mice. For the mice inoculated with atypical scrapie isolates (groups 1a and 1b), a model was used to assess the effect of the PrP^c^ expression levels (a continuous variable) and the prion protein genotype of the animal that was the source of the infectious agent, at 2 factor levels: high susceptibility to atypical scrapie (group 1a: animals with AF_141_RQ or AHQ *prnp* alleles) and low susceptibility to atypical scrapie (group 1b: animals without AF_141_RQ or AHQ *prnp* alleles) [14], [15]. The interaction between the PrP^c^ expression level and the PrP^c^ genotype was tested with the likelihood ratio test.

A Cox model was also applied to the mice inoculated with classical usual scrapie isolates, “CH1641-like” scrapie isolates, experimental TSEs isolates and mouse-adapted strains, to assess the sole effect of PrP^c^ expression level. Survival period was computed as the period between inoculation and death. Proportional hazard assumptions were checked for all models by correlating the corresponding set of scaled Schoenfeld residuals with a suitable transformation of time as proposed by P. Grambsch & al. [70]. The statistical analysis was performed with R software (R version 2-6.0 (2007-11-03): A language and environment for statistical computing. R Foundation for Statistical Computing, Vienna, Austria. ISBN 3-900051-07-0, URL http://www.R-project.org).

### Western blot analysis of PrP^res^

Brain samples were examined by TeSeE WB (Bio-Rad) following the manufacturer's recommendations, as previously described [15]. Briefly after PrP^res^ extractions from 20% brain homogenates and denaturation in Laemmli solution completed with 5% (v/v) β-mercaptoethanol and 2% (w/v) sodium dodecyl sulfate (SDS), the electrophoretic separation was obtained using 15% (resolving gel) and 4% (stacking gel) home-made acrylamide SDS-PAGE gels (200 V, 60 min). After transferring the proteins onto a PVDF membrane (115 V, 60 min), the membranes were incubated 30 min at room temperature with monoclonal antibodies including Sha 31 (4 µg/ml in PBST) [71], P4 (1/2500 in PBST) (R-Biopharm), 4F2 (1/2500 in PBST) and 99/97.6.1 (1/2500 in PBST) (kindly provided by K. O'Rourke, USDA, Pullmann, USA) that recognized the ovine sequences YEDRYYRE (148–155), WGQGGSH (93–99), QPHGGGW (62–93) and YQRE (221–224) (J. Langeveld, unpublished Pepscan data) respectively. The membranes were then incubated with peroxidase-labeled conjugates against mouse immunoglobulins (1/2500 in PBST) (Ozyme), except for the Sha 31 antibody which was revealed with TeSeE WB (Bio-Rad) according to the manufacturer's recommendations. The ECL Western Blotting detection system by enhanced chemiluminescent reaction (ECL, Amersham or Supersignal, Pierce) was then applied. Images were analysed using the Versa Doc analysis system (Bio-Rad) and Quantity One® software (Bio-Rad) [15]. Some experiments involved deglycosylation experiments with PNGase F following the Ozyme manufacturer's instructions (P0704S). After PrP**^res^** purification, the supernatants were treated with PNGase F for 1 h at 37°C (reaction buffer 1X, 10% NP-40, PNGase F), then heated at 100°C for 5 min before Western blot analysis.

### PrP^sc^ immunohistochemistery

Fixed brains were routinely embedded in paraffin after a 1 hour formic acid (98%–100%) treatment at room temperature. De-waxed and re-hydrated 5 µm brain sections were immunostained for PrP^sc^ using SAF84 (SPI Bio) and 2G11 (Pourquier) monoclonal antibodies with or without an additional step using streptomycin sulfate, following a procedure reported in detail elsewhere [72]. A peroxidase-labeled avidin-biotin complex (Vectastain Elite ABC, Vector Laboratories) was used to amplify the signal. Final detection was achieved using a solution of diaminobenzidine intensified with nickel chloride (Zymed), producing black deposits.
